# Arbeitsbezogenes sedentäres Verhalten: Eine explorative Studie an Verwaltungsangestellten einer Universitätsklinik

**DOI:** 10.1007/s40664-022-00489-3

**Published:** 2023-01-09

**Authors:** Paulus Nöscher, Andrea Weber, Michael Leitzmann, Joachim Grifka, Carmen Jochem

**Affiliations:** 1 Institut für Epidemiologie und Präventivmedizin, Universität Regensburg, Franz-Josef-Strauß-Allee 11, 93053 Regensburg, Deutschland; 2 Abteilung für Orthopädie, Universitätsklinikum Regensburg, Asklepios Klinikum Bad Abbach, Kaiser-Karl-V.-Allee 3, 93077 Bad Abbach, Deutschland

**Keywords:** Sedentäres Verhalten, Sitzzeit, Arbeitsmedizin, Public Health, Gesundheitsverhalten, Sedentary behavior, Sitting time, Occupational health, Public health, Health behavior

## Abstract

**Hintergrund:**

Sedentäres Verhalten ist mit einem erhöhten Risiko für chronische Krankheiten und einer höheren Gesamtmortalität assoziiert. Arbeitsbezogenes Sitzen hat einen großen Anteil am täglichen sedentären Verhalten, insbesondere bei Büroangestellten.

**Ziel der Arbeit:**

In dieser Studie sollte untersucht werden, wie viel Zeit bei verschiedenen Aufgaben am Arbeitsplatz und beim Pendeln von Verwaltungsangestellten einer Universitätsklinik in Deutschland im Sitzen verbracht wird.

**Material und Methoden:**

Eine fragebogengestützte Querschnittsstudie mit Verwaltungsangestellten des Universitätsklinikums Regensburg wurde durchgeführt, um arbeitsbezogenes sedentäres Verhalten zu untersuchen.

**Ergebnisse:**

Die Studienpopulation bestand aus 159 Teilnehmenden (54,1 % Frauen, 51,6 % älter als 40 Jahre), was einer Antwortquote von 26 % entspricht. Die durchschnittliche tägliche Sitzzeit am Arbeitsplatz betrug im Median 7,0 h (Interquartilsbereich [IQR] 6,0–7,5 h) und fand hauptsächlich bei der Computerarbeit statt (57,3 %). Die mittlere Stehzeit bei der Arbeit betrug im Median 0,8 h (IQR 0,3–1,4 h). Verwaltungsangestellte verbrachten während des Pendelns im Median 0,7 h (IQR 0,3–1,0 h) pro Tag im Sitzen. Die Teilnehmenden waren der Ansicht, dass langes und ununterbrochenes Sitzen negative (69,6 %) oder eher negative (29,7 %) Auswirkungen auf die Gesundheit hat.

**Diskussion:**

Verwaltungspersonal in Krankenhäusern verbringt einen großen Teil der täglichen Arbeitszeit mit sedentärem Verhalten. Maßnahmen, die es ermöglichen, sowohl im Sitzen als auch im Stehen zu arbeiten, können zu einer Verringerung der arbeitsbedingten Sitzzeit führen und damit die Gesundheit am Arbeitsplatz und im weiteren Sinne die öffentliche Gesundheit verbessern.

**Zusatzmaterial online:**

Zusätzliche Informationen sind in der Online-Version dieses Artikels (10.1007/s40664-022-00489-3) enthalten.

## Abstract (en)

**Background:**

Sedentary behavior is associated with an increased risk of chronic diseases, such as metabolic syndrome and type 2 diabetes and all-cause mortality. Occupational sitting time contributes to large amounts of daily sedentary behavior, especially in office workers.

**Objective:**

This study investigated the amount of time spent in sedentary behavior during different tasks at work and while commuting of administrative personnel of a university hospital in Germany.

**Material and methods:**

A cross-sectional questionnaire-based study of administrative employees at the University Hospital Regensburg, Germany was carried out to assess work-related sedentary behavior. Descriptive and exploratory statistical analyses were performed.

**Results:**

The study population consisted of 159 participants (54.1% women, 51.6% older than 40 years), which corresponds to a response rate of 26%. The median daily sitting time in the office was 7.0 h (interquartile range, IQR 6.0–7.5 h) and mostly occurred during computer work (57.3%), telephone calls (13.2%) and meetings (11.7%). Median standing time at work was 0.8 h (IQR 0.3–1.4 h). Administrative staff spent a median of 0.7 h (IQR 0.3–1.0 h) per day sedentary while commuting, with 67.3% of respondents commuting by car, motorbike or scooter. The participants were of the opinion that sitting for long uninterrupted periods had negative (69.6%) or relatively negative (29.7%) effects on health.

**Conclusion:**

Administrative staff in hospitals spend large amounts of the daily working time with sedentary behavior. Interventions that enable working both in sitting and standing positions can lead to reduced work-related sitting time and thereby could improve occupational and, in a broader sense, public health.

Sedentäres Verhalten ist definiert als „jegliche Art von Verhalten, die im Wachzustand in einer Sitz‑, Liegesitz- oder Liegeposition und bei einem Energieverbrauch von ≤ 1,5 Metabolischen Äquivalenten (METs) durchgeführt wird“ [37]. Bei berufstätigen Erwachsenen sind Arbeit, Freizeit und Fortbewegung die Hauptbereiche, die zum täglichen sedentären Verhalten beitragen [7, 25]. Die durchschnittliche Sitzdauer von Erwachsenen in Deutschland liegt bei 8,5 h pro Tag und wird durch den sozioökonomischen Status, das Alter, das Geschlecht und andere Faktoren beeinflusst [15]. Die Prävalenz von sedentärem Verhalten während des Pendelns wurde in Deutschland noch nicht untersucht, aber 68,6 % nutzen mit PKW oder Motorrad ein zwingend sedentäres Fortbewegungsmittel [30]. In Deutschland verbringen Erwachsene, die an einem Schreibtisch arbeiten, 73,0 % ihrer Arbeitszeit mit sitzenden Tätigkeiten [38].

Seit Jahren gibt es einen wissenschaftlich erwiesenen Zusammenhang zwischen einem hohen Maß an sedentärem Verhalten und einem erhöhten Risiko für die Gesamtmortalität und negative gesundheitliche Effekte, wie metabolisches Syndrom, Typ-2-Diabetes, verschiedene Krebsarten und Rücken‑/Nackenschmerzen, die unabhängig von körperlicher Aktivität sind [4, 9, 13, 14, 19, 28, 29, 40].

In den jüngsten Leitlinien der Weltgesundheitsorganisation (WHO) zu körperlicher Aktivität und sedentärem Verhalten sowie im Globalen Aktionsplan für körperliche Aktivität der WHO wird der Arbeitsplatz als wichtiger Ort für Interventionen genannt [41, 43]. Empfehlungen für Interventionen, die einen bewegungsreichen Arbeitsablauf fördern, sind für Deutschland von der Bundesanstalt für Arbeitsschutz und Arbeitsmedizin (BAUA) und der Deutschen Gesetzlichen Unfallversicherung (DGUV) verfügbar [1, 27]. In einer aktuellen Metaanalyse zeigte eine höhere persönliche Gesundheitskompetenz, dazugehörend das Wissen über die Folgen persönlichen Verhaltens auf die Gesundheit, auch eine höhere körperliche Aktivität und niedrigeres sedentäres Verhalten [8]. Die Senkung der hohen Prävalenz von sedentärem Verhalten ist nicht nur eine wichtige Präventionsstrategie für die Gesundheit jedes Einzelnen, sondern auch im Hinblick auf Public Health, da chronische Krankheiten die Haupttodesursachen in Deutschland sind [24]. Darüber hinaus ist die Stärkung der Gesundheitsförderung beim medizinischen und nichtmedizinischen sowie administrativ tätigen Personal von Krankenhäusern Teil der Bemühungen, um grüne und gesunde Krankenhäuser in Deutschland zu fördern [2, 17].

Die vorliegende Studie zielt darauf ab, den Umfang und den Kontext der arbeitsbedingten Sitz- und Stehzeiten von Verwaltungsangestellten einer Universitätsklinik in Deutschland zu untersuchen, einschließlich der sitzenden Tätigkeit bei der Arbeit und beim Pendeln. Darüber hinaus soll mit dieser Studie das vorhandene Wissen der Teilnehmenden über die Auswirkungen von sedentärem Verhalten auf die Gesundheit, den Einfluss des Vorgesetzten auf das sedentäre Verhalten und gewünschte potenzielle Interventionen zur Reduktion von arbeitsbedingtem sedentärem Verhalten untersucht werden.

## Methode

Im Juni 2021 führten wir eine fragebogengestützte Querschnittsstudie zu Ergonomie und arbeitsbedingtem sedentärem Verhalten bei Verwaltungsangestellten des Universitätsklinikums Regensburg (*n* = 600) durch. Alle Teilnehmenden gaben ihr Einverständnis zur anonymen und freiwilligen Datenerhebung. Das Studienprotokoll wurde von der Ethikkommission der Universität Regensburg unter der Verfahrensnummer 21-2287-104 genehmigt.

Ein deutschsprachiger Online-Fragebogen mit 38 Items wurde mit mehreren modifiziert übertragenen Fragen aus den im Folgenden genannten validierten englischsprachigen Fragebögen entworfen (Onlinematerial 1) und in LimeSurvey Professional (Version 3.27.16) implementiert [21]. Aus dem Workforce Sitting Questionnaire (WSQ) wurde die Frage zur Sitzzeit modifiziert übertragen. Der WSQ zeigte eine mittel bis gute Test-Retest-Reliabilität (Intraklassen-Korrelationskoeffizient 0,63; 95 % KI 0,49–0,74) und eine niedrige Validität (Spearman’s Korrelationskoeffizient 0,45; *p* < 0,01) im Vergleich zu Akzelerometer-bestimmtem arbeitsbezogenem sedentärem Verhalten. Allerdings wiesen Bland-Altman-Diagramme darauf hin, dass der WSQ im Vergleich zu Akzelerometer-bestimmtem arbeitsbezogenem sedentärem Verhalten eine Unterschätzung bei niedrigen gemessenen Sitzzeiten und eine Überschätzung bei höheren gemessenen Sitzzeiten zeigte [10]. Im Vergleich zum häufig verwendeten International Physical Activity Questionnaire (IPAQ) zeigte der WSQ eine mäßig starke Korrelation (Spearman’s Korrelationskoeffizient 0,59; *p* < 0,01; [10]). Die Frage zu Sitzunterbrechungen wurde aus der Stand Up Australia Studie modifiziert übertragen. Dort konnte ein statistisch signifikanter Zusammenhang zwischen den selbstberichteten und den mittels Akzelerometer gemessenen Sitzunterbrechungen bei Büroangestellten (Korrelationskoeffizient 0,23, 95 % KI 0,02–0,43) gezeigt werden [11]. In der vorliegenden Studie wurde die Zeit, die im Sitzen verbracht wurde, mit Schiebereglern erfasst, und es wurden Plausibilitätsprüfungen eingebaut, um die Validität der Antworten zu verbessern.

Soziodemografische Informationen zu Alter, Geschlecht, Bildungsstand (niedrige und andere Bildung [kein oder anderer Abschluss, Sekundarschulabschluss], mittlere Bildung [Hochschulreife, Berufsausbildung] und hohe Bildung [Universitätsabschluss]), und Body-Mass-Index (BMI) wurden erhoben. Der BMI wurde anhand der Grenzwerte der WHO kategorisiert: < 18,5 kg/m^2^ als „Untergewicht“, 18,5–24,9 kg/m^2^ als „gesundes Gewicht“, 25–29,9 kg/m^2^ als „Übergewicht“ und ≥ 30 kg/m^2^ als „Adipositas“ [42].

Deskriptive und explorative statistische Analysen wurden mit der Statistiksoftware R, Version 4.0.5 durchgeführt [35]. Für kategoriale Daten wurden Häufigkeiten und Proportionen berechnet. Für kontinuierliche Daten wurden Mittelwerte mit Standardabweichungen berechnet und bei Abweichungen von der Normalverteilung der Median mit 25. und 75. Quantile.

## Ergebnisse

### Demografische Merkmale

Die Studienpopulation bestand aus 159 Teilnehmenden (54,1 % Frauen, 51,6 % älter als 40 Jahre), was einer Antwortquote von 26 % entspricht. Ein Flussdiagramm der Teilnehmenden ist im Onlinematerial 2 zu finden. Tab. 1 zeigt die demografischen Merkmale der Gesamtstichprobe.

**Table 1:** 

	Frauen (*n* = 86)	Männer (*n* = 73)
*Alter (Jahre)*		
18–30	27	11
31–40	23	16
41–50	14	17
> 50	22	29
*BMI (kg/m*^*2*^*)*		
Untergewicht (< 18,5)	5	1
Gesundes Gewicht (18,5–24,9)	54	31
Übergewicht (25–29,9)	20	23
Adipositas (≥ 30)	7	18
*Bildungsniveau*		
Niedrig und andere	19	16
Mittel	25	23
Hoch	42	31
	**Median (25.–75. Perzentile)**	**Median (25.–75. Perzentile)**
*Wöchentliche Arbeitsstunden*	38,5 (35,0–40,0)	39,0 (38,5–40,0)
*Tägliche Arbeitsstunden (min)*	8,0 (7,3–8,5) (480 [465–510])	8,3 (8,0–8,5) (495 [480–510])
*Tägliche Sitzzeit am Arbeitsplatz (min)*	7,0 (6,0–7,5) (420 [360–450])	7,0 (5,3–7,5) (420 [315–450])
*Tägliche Stehzeit am Arbeitsplatz (min)*	0,5 (0,25–1,0) (30 [15–60])	1,0 (0,5–2,0) (60 [30–120])

### Sitz- und Stehgewohnheiten bei der Arbeit

Die wöchentliche und tägliche Arbeitszeit sowie die tägliche Sitz- und Stehzeit am Arbeitsplatz sind in Tab. 1 aufgeführt. An einem typischen Tag verbrachte das Verwaltungspersonal im Durchschnitt 8,1 h bei der Arbeit (Standardabweichung [SD] = 0,9 h; Median = 8,0 h), wobei Männer (Mittelwert = 8,2 h; SD = 0,6 h; Median = 8,3 h) etwas länger arbeiteten als Frauen (Mittelwert = 8,0 h; SD = 1,1 h; Median = 8,0 h).

**Figure 1:**
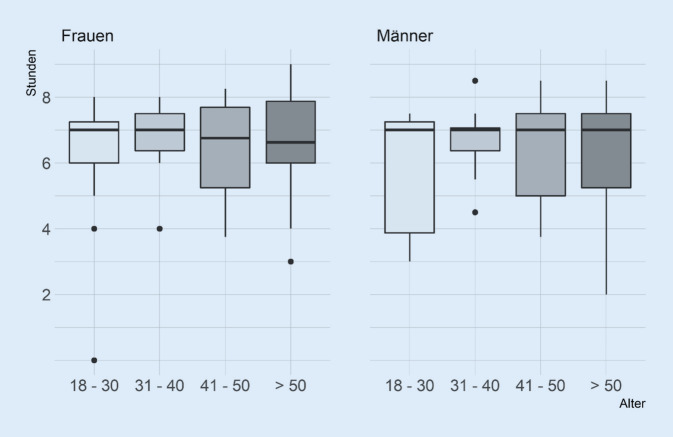

Der Median der gesamten täglichen Sitzzeit betrug 7,0 h (IQR 6,0–7,5 h; Mittelwert = 6,5 h; SD = 1,5 h). Die Teilnehmenden verbrachten im Durchschnitt 80,0 % (SD = 16,8 %) ihrer Arbeitszeit in sitzender Position, wobei es große interindividuelle Unterschiede gab. Frauen verbrachten 82,2 % ihres Büroarbeitstages (SD = 16,0 %; Median = 86,5 %) mit sedentärem Verhalten, verglichen mit 77,4 % bei Männern (SD = 17,5 %; Median = 83,9 %). Die Stundenzahl, die bei der Arbeit im Sitzen verbracht wurde, in Abhängigkeit von Alter und Geschlecht, zeigt Abb. 1. Die Zeit, die im Sitzen verbracht wurde, war in den verschiedenen BMI-Kategorien ähnlich, wobei der Median zwischen 79,0 % (Mittelwert = 70,6 %; SD = 24,3 %) bei untergewichtigen und 87,5 % (Mittelwert = 80,6 %; SD = 15,6 %) bei adipösen Teilnehmenden lag. Befragte mit niedrigem Bildungsniveau (Mittelwert = 75,6 %; sd = 17,8 %; Median = 80,0 %) verbrachten etwas weniger Zeit mit sitzenden Tätigkeiten während der Arbeit als Teilnehmende mit mittlerem (Mittelwert = 75,7 %; SD = 20,5 %; Median = 81,1 %) und hohem Bildungsniveau (Mittelwert = 84,8 %; SD = 11,9 %; Median = 87,5 %).

Die Ergebnisse zeigten große interindividuelle Unterschiede (0,0–6,5 h) bei der beruflichen Stehzeit, bei einem Median von 0,8 h (IQR 0,3–1,4 h; Tab. 1). Männer verbrachten im Durchschnitt 15,7 % ihrer Arbeitszeit im Stehen (SD = 15,8 %; Median = 12,1 %), Frauen 10,7 % (SD = 12,0 %; Median = 7,1 %). Die Zeit, die im Stehen verbracht wurde, war in den verschiedenen BMI-Kategorien ähnlich, wobei der Median zwischen 9,0 % (Mittelwert = 11,0 %; SD = 11,9 %) bei untergewichtigen und 11,1 % (Mittelwert = 13,6 %; SD = 11,8 %) bei adipösen Teilnehmenden lag. Befragte mit niedrigem Bildungsniveau verbrachten einen größeren Anteil ihrer Arbeitszeit im Stehen (17,5 %; SD = 17,7 %; Median = 12,5 %) im Vergleich zu Teilnehmenden mit mittlerem (14,7 %; SD = 15,5 %; Median = 11,9 %) oder hohem Bildungsniveau (9,9 %; SD = 10,3 %; Median = 6,3 %). In absoluten Zahlen verbrachte die Gruppe mit mittlerer Schulbildung 27,6 min pro Tag mehr Zeit mit stehenden Tätigkeiten als die Gruppe mit hoher Schulbildung.

**Figure 2:**
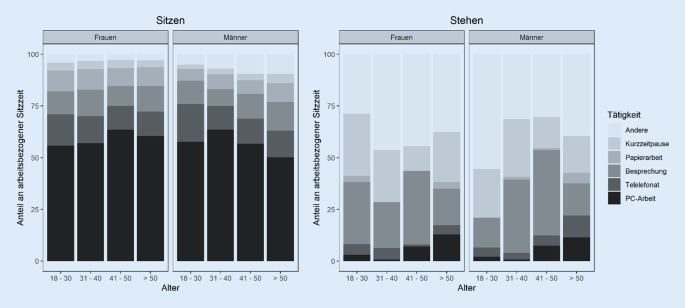

Die verschiedenen Tätigkeiten, die im Stehen bzw. Sitzen ausgeführt wurden, sind in Abb. 2 dargestellt. Im Durchschnitt trug die Arbeit am Computer zu 57,3 % (SD = 18,6 %) der sitzenden Arbeitszeit bei, gefolgt von Telefonaten (13,2 %; SD = 9,5 %), Besprechungen (11,7 %; SD = 8,5 %), Papierarbeit (8,6 %; SD = 8,3 %), sonstigen Tätigkeiten (5,6 %; SD = 8,8 %) und Kurzzeitpausen (3,7 %; SD = 3,8 %).

Bei den Tätigkeiten im Stehen ergibt sich ein heterogeneres Bild zwischen den Altersgruppen und Geschlechtern als bei den Tätigkeiten im Sitzen (Abb. 2). Die Teilnehmenden gaben an, dass sonstige Tätigkeiten (38,2 %; SD = 35,8 %), Besprechungen (25,7 %; SD = 28,9 %) und kurze Pausen (22,5 %; SD = 30,7 %) den größten Anteil der im Stehen verbrachten Arbeitszeit ausmachten, gefolgt von Computerarbeit (5,6 %; SD = 8,8 %), Telefonaten (5,4 %; SD = 9,2 %) und Papierarbeit (2,1 %; SD = 6,5 %).

Die meisten Befragten gaben an, einmal pro Stunde aufzustehen, um das Sitzen zu unterbrechen (*n* = 38; 23,9 %), während 36 (22,64 %) Teilnehmende das Sitzen zweimal, 29 (18,24 %) dreimal, 20 (12,58 %) viermal und 29 (18,24 %) fünfmal oder häufiger ihr Sitzen pro Stunde unterbrachen. Sieben Teilnehmende (4,4 %) unterbrachen das Sitzen während einer Stunde nicht.

Die COVID-19-Pandemie und die damit verbundenen Einschränkungen hatten bei der Mehrheit der Studienpopulation keinen Einfluss auf die Sitzdauer (67,5 %) und die Häufigkeit der Sitzunterbrechungen (74,1 %). Ein Drittel der Teilnehmenden (31,8 %) verbrachte mehr Zeit im Sitzen bei der Arbeit und ein Fünftel (20,9 %) unterbrach sein sedentäres Verhalten weniger häufig.

### Vorwissen über die Auswirkungen sedentären Verhaltens auf die Gesundheit

**Figure 3:**
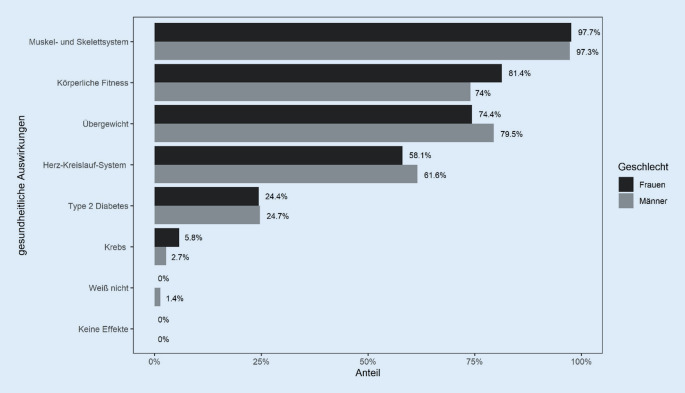

Fast alle Befragten waren der Ansicht, dass langes und ununterbrochenes sedentäres Verhalten negative (*n* = 110; 69,6 %) oder eher negative (*n* = 47; 29,7 %) Auswirkungen auf die menschliche Gesundheit hat. Vorwissen über die Effekte von sedentärem Verhalten auf verschiedene Gesundheitsaspekte war unter den Befragten besonders in den Bereichen Muskel- und Skelettsystem (*n* = 155; 97,5 %), körperliche Fitness (*n* = 124; 78,0 %) und Übergewicht (*n* = 122; 76,7 %) vorhanden. Auswirkungen auf das Herz-Kreislauf-System (*n* = 95; 59,7 %) und Typ-2-Diabetes (*n* = 39; 24,5 %) waren seltener bekannt, wobei ein Zusammenhang mit Krebs nur von 7 Teilnehmenden (4,4 %) genannt wurde. In Abb. 3 sind die Ergebnisse stratifiziert nach Geschlecht dargestellt. Die Ergebnisse waren über alle Bildungsniveaus hinweg ähnlich, wie in Onlinematerial 3 dargestellt.

### Rolle der Vorgesetzten für das arbeitsbezogene sedentäre Verhalten

Die Mehrheit der Befragten ging davon aus, dass ihre Vorgesetzten eine neutrale (*n* = 79; 51,3 %), eher positive (*n* = 24; 15,6 %) oder positive (*n* = 24; 15,6 %) Einstellung zum Stehen am Arbeitsplatz haben, während 9 (5,8 %) von einer eher negativen Einstellung ausgingen und 18 (11,7 %) es nicht wussten.

Teilnehmende, die davon ausgingen, dass ihre Vorgesetzten das Stehen bei der Arbeit gutheißen würden, verbrachten einen geringeren Anteil ihrer Arbeitszeit im Stehen (10,5 %; SD = 10,7 %; Median = 8,4 %) als die übrigen Befragten (13,0 %; SD = 14,1 %; Median = 10,0 %).

### Interventionswünsche zur Verringerung von arbeitsbedingtem sedentärem Verhalten

**Figure 4:**
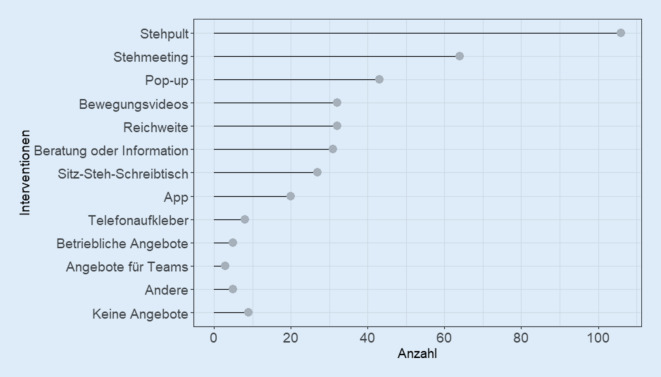

Auf die Frage nach den gewünschten Interventionsmöglichkeiten zur Verringerung von sedentärem Verhalten am Arbeitsplatz sprachen sich die meisten Befragten für Stehpulte aus (*n* = 106; 66,7 %). Einzelheiten zu den gewünschten Interventionen sind in Abb. 4 dargestellt. Stehmeetings (*n* = 64; 40,3 %) wurden am häufigsten in der Altersgruppe der 41- bis 50-Jährigen (*n* = 17; 51,6 %) und häufiger von Teilnehmenden mit hohem Bildungsniveau (*n* = 38; 52,1 %) gewählt. Details zu den Wünschen der Teilnehmenden nach Altersgruppen und Bildungsniveaus sind in Onlinematerial 4 und 5 dargestellt.

### Pendeln von und zur Arbeit

**Figure 5:**
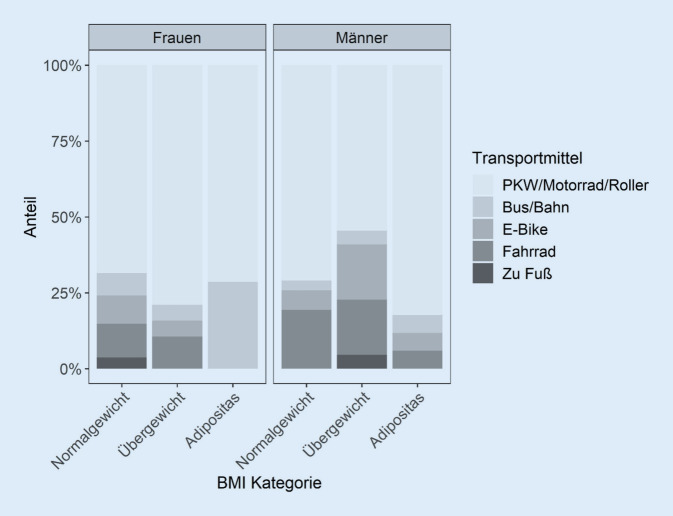

Die Mehrheit (*n* = 118; 73,6 %) der Befragten nutzte im Sommer Verkehrsmittel, die mit sedentärem Verhalten einhergehen, nämlich Auto, Motorrad oder Motorroller (*n* = 107; 67,3 %) oder öffentliche Verkehrsmittel (*n* = 11; 6,9 %). Aktive Fortbewegungsmittel wurden von 34 (21,3 %) Teilnehmenden genutzt, von denen 21 (13,2 %) mit dem Fahrrad zur Arbeit kamen und 13 (8,2 %) ein E‑Bike nutzten. Autos, Motorräder und Motorroller wurden in der Gruppe der 50-Jährigen und Älteren am wenigsten genutzt, unabhängig vom Geschlecht (Abb. 5). Bei Frauen stieg der Anteil der Verkehrsmittel, die mit sedentärem Verhalten einhergehen, mit zunehmendem BMI.

Die durchschnittliche Dauer eines einfachen Pendelwegs betrug 31,3 min (SD = 22,9 min; Median = 30,0 min), wovon im Durchschnitt 23,3 min im Sitzen verbracht wurden (SD = 22,1 min; Median = 20,0 min). Im Median wurden 0,7 h pro Tag (IQR 0,3–1,0 h) während des Pendelns im Sitzen verbracht.

## Diskussion

Ziel dieser Studie war es, das sedentäre Verhalten bei der Arbeit und beim Pendeln in einer Stichprobe von Krankenhausverwaltungsangestellten in Deutschland zu untersuchen und einige Determinanten der arbeitsbedingten sitzenden Tätigkeit zu untersuchen sowie die gewünschten Maßnahmen zur Verringerung des arbeitsbedingten sedentären Verhaltens zu ermitteln. Die Ergebnisse der Studie zeigen, dass die durchschnittliche Zeit, die mit sitzenden Tätigkeiten verbracht wurde, im Median 7,0 h pro Tag (IQR 6,0–7,5 h) während der Arbeit und im Median 0,7 h pro Tag (IQR 0,3–1,0 h) beim Pendeln betrug.

Unsere Ergebnisse stehen im Einklang mit den Resultaten mehrerer anderer Studien, die sedentäres Verhalten bei Büroangestellten untersuchten. Wallmann-Sperlich et al. zeigten zum Beispiel, dass Büroangestellte in Deutschland 73,0 % (SD = 21,7 %) ihrer täglichen Arbeitszeit sedentär verbringen [38]. Studien aus anderen Ländern ergaben arbeitsbezogene Sitzzeiten zwischen 5,5 und 6,0 h pro Tag [26, 33]. Bei der beruflichen Stehzeit zeigten sich große interindividuelle Unterschiede (0,0–6,5 h pro Tag), wobei unklar blieb, ob dies durch die Tätigkeit (etwa am Empfang) oder bewusstes gesundheitsförderliches Verhalten des Mitarbeitenden oder durch andere Gründe zu erklären ist. Das Ergebnis, das Teilnehmende, die davon ausgingen, dass ihre Vorgesetzten das Stehen bei der Arbeit begrüßen würden, einen geringeren Anteil ihrer Arbeitszeit im Stehen verbrachten, war mit den vorliegenden Daten nicht zu erklären. Möglicherweise könnte dies durch den psychologischen Effekt der Reaktanz, also die Motivation zur Wiederherstellung eingeengter Freiheitsspielräume, erklärt werden [31]. Andere mögliche einflussnehmende Faktoren könnten das Verhalten und die Einstellungen der Kolleginnen und Kollegen, die Arbeitsorganisation sowie bereits vorhandene, das Stehen fördernde, individuelle Maßnahmen im Arbeitsbereich sein.

In der vorliegenden Studie trugen Computerarbeit, Telefongespräche und Papierarbeit am eigenen Schreibtisch zum größten Teil der sedentären Tätigkeiten am Arbeitsplatz bei. Diese sitzenden Tätigkeiten am Arbeitsplatz könnten ein potenzielles Ziel für Maßnahmen zur Verringerung von sedentärem Verhalten im beruflichen Kontext sein. Die von den Teilnehmenden gewünschten Interventionen betreffen teilweise diese Bereiche und stehen im Einklang mit den Empfehlungen der BAUA. So stimmt der Wunsch nach Stehmeetings, die Möglichkeit der Aufsteherinnerung (App und Pop-up) und die Möglichkeit im Stehen zu Arbeiten (Stehpult und Sitz-Steh-Schreibtisch) mit den Empfehlungen überein [1]. Weitere Empfehlungen der DGUV, wie das Aufstellen von Druckern außerhalb der Arbeitsplatzreichweite oder Angebote für Gruppen, wurden von den Mitarbeitenden nur wenig gewünscht [27]. Eine aktuelle Übersichtsarbeit zeigte, dass Maßnahmen, die auf die physische Umgebung abzielen (z. B. Sitz-Steh-Tische), am wirksamsten zur Verringerung der sitzenden Tätigkeit im Büro sind und die sitzende Tätigkeit um 87,9 min pro Tag reduzieren [20]. Dies passt gut zu den Wünschen der Befragten nach Stehmeetings, Stehpulten und Sitz-Steh-Schreibtischen. Obwohl diese Maßnahmen mit Kosten verbunden sind, zeigen Kosten-Nutzen-Analysen Netto-Kosteneinsparungen [20, 23]. Gao et al. [16] zeigten für Steh-Sitz-Schreibtische eine subjektive Verbesserung des Muskel-Skelett-Systems im Bereich des Nackens und der Schultern. Im Rahmen von Stand Up Victoria konnte mit einer Intervention, bestehend aus den Bereichen Unternehmensführung (Unterstützung durch die Geschäftsleitung, Rekrutierung eines Team-Champions), einer Setting Komponente (Steh-Sitz-Arbeitsplätze) und einer individuellen Komponente (Gesundheitsberatung, Zielsetzung und Nachverfolgung), eine positive Veränderung der metabolischen Situation gezeigt werden [18]. Eine alternative und kostengünstigere Möglichkeit, die Arbeitshöhe für stehende Arbeit anzupassen, könnte die Verwendung von geeigneten Schreibtischaufsätzen sein. Zusätzlich zu den Maßnahmen im Büro bieten Stehmeetings eine weitere Möglichkeit, sedentäres Verhalten am Arbeitsplatz zu reduzieren. Dies steht im Einklang mit den Ergebnissen von Stand Up Lendlease und Stand Up Victoria [6, 32].

In der vorliegenden Studie nutzte die Mehrheit der Befragten ein Verkehrsmittel, welches mit sedentärem Verhalten einhergeht, für den Weg von oder zur Arbeit. Gründe hierfür könnten sein, dass das Universitätsklinikum am Stadtrand von Regensburg auf einer Anhöhe liegt, ein naher Autobahnanschluss besteht und Busse die einzige Möglichkeit des öffentlichen Personennahverkehrs darstellen. Diese besonderen (topografischen) Bedingungen könnten den hohen Anteil an Autos, Motorrädern und Motorrollern und die geringe Anzahl an Fahrrädern bei der Transportmittelwahl beeinflusst haben. In einer australischen Studie wurde ein Median von 60 min für sitzende Fortbewegung pro Tag ermittelt, und eine taiwanesische Studie ergab einen Mittelwert von 81,55 min sedentären Verhaltens während des Transports (SD = 98,39 min; [3, 22]). Kürzlich veröffentlichte Metaanalysen zeigten einen positiven Zusammenhang zwischen aktivem Pendeln und einer geringeren Gesamt- und kardiovaskulären Sterblichkeit sowie ein geringeres Risiko für die Entwicklung von Adipositas, Bluthochdruck und Diabetes [12, 44]. Somit ist aktives Pendeln als Interventionsmöglichkeit, insbesondere zur Reduktion der Gesamtsitzzeit eines Tages, zu werten. Der Einfluss des Arbeitgebers, neben den Rahmenbedingungen am direkten Arbeitsplatz, kann durch die Schaffung von sicheren Fahrradstellplätzen und ausreichenden Umkleiden/Duschen zu einer höheren Quote an aktiv Pendelnden beitragen [45]. Maßnahmen, die eine aktive Fortbewegung von oder zur Arbeit ermöglichen, wie z. B. Firmenrad-Leasing-Programme, könnten die Gesundheit von Arbeitnehmern verbessern [34].

Die meisten Teilnehmenden waren sich der negativen gesundheitlichen Auswirkungen des Sitzens auf den Bewegungsapparat, die Fitness und das Übergewicht bewusst und sahen die Notwendigkeit von Maßnahmen. Allerdings fehlte es an Wissen zu den negativen Effekten von langem und ununterbrochenem Sitzen in Bezug auf die Entwicklung von häufigen chronischen Erkrankungen wie Typ-2-Diabetes und Krebs, die ins Bewusstsein gerufen werden müssen, um eine Verhaltensänderung zu ermöglichen. Hier können Informationskampagnen am Arbeitsplatz einen Beitrag leisten, wie beispielsweise „Move More, Sit Less“ in Australien [5].

Die Stärken der vorliegenden Studie sind die detaillierte Datenerhebung zum Kenntnisstand über die gesundheitlichen Folgen des sedentären Verhaltens sowie die Erhebung der Interventionswünsche der Verwaltungsangestellten. Darüber hinaus wurden Informationen über bereichsspezifische sedentäre Zeiten gesammelt, da der Forschungsstand hierzu unzureichend ist. Durch den Einsatz eines Online-Befragungsinstruments konnten wir die Gefahr für unplausible Daten reduzieren. So konnte beispielsweise die eingegebene Sitzzeit nicht die abgefragte Arbeitszeit übersteigen, auch wurden harte Plausibilitätsgrenzen gesetzt. Zudem hat die Studienpopulation in der Vergangenheit keine spezifische Intervention zur Verringerung des sedentären Verhaltens erhalten und hat daher ein hohes Potenzial für Interventionen.

Die vorliegende Studie weist jedoch auch einige Einschränkungen auf. Die Antwortquote ist im Vergleich zu anderen Online-Mitarbeiterbefragungen eher gering. Thielsch und Weltzin [36] beschreiben eine zu erwartende Rücklaufquote zwischen 60 und 80 %, abhängig von Faktoren wie der begleitenden Kommunikationskampagne. Im vorliegenden Fall wurden neben Plakaten auch die Abteilungsleitenden telefonisch informiert, Mails zur Weiterleitung an alle Mitarbeitenden zur Verfügung gestellt sowie als Erinnerung persönliche Gespräche mit den Abteilungsleitenden geführt. Die COVID-19-Pandemie und die damit verbundenen Einschränkungen und höhere Arbeitsbelastung könnten ein weiterer Grund für die niedrige Antwortquote sein. Die Verwendung eines subjektiven Instrumentes zur Erfassung des sedentären Verhaltens ist anfällig für Antwortverzerrungen aufgrund von sozialer Erwünschtheit oder Erinnerungsschwierigkeiten. Jedoch berichten Warren et al. [39], dass Online-Fragebögen ein größeres Gefühl von Anonymität bei den Befragten erzeugt und somit zu einer valideren Angabe sensibler Informationen führt. Weitere Vorteile sind, dass Online-Fragebögen leicht standardisierbar sind und die Möglichkeit bieten, Erklärungen, Aufforderungen, Fehlerkorrekturen, Menüs, Verzweigungen und Überspringen einzusetzen [39]. Online-Fragebögen sind auch zeit- und kostensparend, da die digitalen Daten schnell statistisch ausgewertet werden können und Kodierungsfehler vermieden werden [39]. Auf die Verwendung gerätebasierter Methoden, wie am Oberschenkel getragener Akzelerometer, wurde in diesem Setting verzichtet, da durch diese keine Kontextinformationen ermittelt werden können, welche jedoch für diese Studie von großer Relevanz sind. Weiterhin ist als limitierend zu betrachten, dass das Verhalten und die Einstellungen der Kolleginnen und Kollegen, die Arbeitsorganisation sowie bereits vorhandene, das Stehen fördernde, individuelle Maßnahmen im Arbeitsbereich vom Fragebogen nicht erfasst wurden. Die fehlende internationale Studienlage, beispielsweise zum Vorwissen bei Verwaltungsangestellten, lässt nur eingeschränkt Vergleiche zu, weshalb die Ergebnisse dieser Studie umso wichtiger sind. Zuletzt kann anhand der hier durchgeführten Studie nicht geschlussfolgert werden, ob sich das Sitz- und Stehverhalten von Verwaltungsangestellten einer Universitätsklinik von Verwaltungsangestellten anderer Sektoren unterscheidet.

Diese Studie zeigt, wie wichtig es ist, zielgruppenspezifische Maßnahmen für Büroangestellte einschließlich des Verwaltungspersonals in Krankenhäusern durchzuführen. Die Verringerung der Zeit, die mit sitzender Tätigkeit verbracht wird, ist ein wichtiger Bestandteil von moderner evidenzbasierter Präventivmedizin und Public Health, um die negativen Auswirkungen von sedentärem Verhalten auf die menschliche Gesundheit zu reduzieren [41, 43]. In der weiteren Forschung sollte die Wirksamkeit der Maßnahmen evaluiert werden.

## Fazit

Unsere Ergebnisse zeigen ein hohes Maß an sitzender Tätigkeit während der Arbeit und während des Pendelns bei Verwaltungsangestellten eines Universitätsklinikums in Deutschland. Die Ergebnisse verdeutlichen das große Potenzial von Maßnahmen, die sedentäre Tätigkeiten und die damit einhergehende Sitzzeit bei Büroangestellten im Gesundheitswesen reduzieren können. Maßnahmen, die das Arbeiten sowohl im Sitzen als auch im Stehen ermöglichen, und ein größeres Wissen über die negativen Effekte von langem Sitzen könnten zu einer Verringerung der arbeitsbedingten Sitzzeiten führen und damit die Gesundheit am Arbeitsplatz und in der Bevölkerung verbessern.

## Supplementary Information




