# Supplementary material for: Work-related sedentary behavior: An exploratory study of administrative staff in a university hospital
Source: Zentralbl Arbeitsmed Arbeitsschutz Ergon. 2023 Jan 9;73(1):39–47. [Article in German] doi: 10.1007/s40664-022-00489-3 (PMC9829228; doi:10.1007/s40664-022-00489-3)
Supplement: Supplementary file 1 [file 40664_2022_489_MOESM1_ESM.pdf]

Onlinematerial

Onlinematerial 1 Fragebogen Ergonomie und Sitzverhalten Universitätsklinikum Regensburg

Jede Frage kann übersprungen werden! Die Fragen zu Ergonomie (Nr. 26 – 32) können aus Urheberrechtsgründen hier nicht veröffentlicht werden.

<table>
<tr><td colspan="13">Sitzverhalten
Allgemein</td></tr>
<tr><td>1.</td><td colspan="12">Ist Ihnen etwas über die gesundheitlichen Auswirkungen übermäßigen Sitzens bekannt?</td></tr>
<tr><td></td><td colspan="6">Ja</td><td colspan="6">Nein</td></tr>
<tr><td>2.</td><td colspan="12">Welche drei Stichpunkte kommen Ihnen zum Thema Sitzzeiten spontan in den Sinn?</td></tr>
<tr><td></td><td colspan="12"></td></tr>
<tr><td>3.</td><td colspan="12">Wie beeinflusst langes ununterbrochenes Sitzen die Gesundheit des Menschen Ihrer Meinung nach?</td></tr>
<tr><td></td><td colspan="2">Negativ</td><td colspan="2">Eher negativ</td><td colspan="2">Neutral</td><td colspan="2">Eher positiv</td><td colspan="2">Positiv</td><td colspan="2">Weiß nicht</td></tr>
<tr><td>4.</td><td colspan="12">Ihrer Meinung nach hat das Sitzverhalten (Dauer, Unterbrechungen, etc.) einen wissenschaftlich nachgewiesenen Effekt auf...
Multiple Choice möglich</td></tr>
<tr><td rowspan="8"></td><td colspan="12">Muskel- und Skelettsystem</td></tr>
<tr><td colspan="12">Körperliche Fitness</td></tr>
<tr><td colspan="12">Diabetes mellitus Typ 2</td></tr>
<tr><td colspan="12">Übergewicht</td></tr>
<tr><td colspan="12">Herz-Kreislauf-System (z.B. Schlaganfall, Herzinfarkt)</td></tr>
<tr><td colspan="12">Krebs (z.B. Darmkrebs, Brustkrebs, Eierstockkrebs)</td></tr>
<tr><td colspan="12">Keine Effekte</td></tr>
<tr><td colspan="12">Weiß nicht</td></tr>
<tr><td colspan="13">Rahmenbedingungen an Ihrem Arbeitsplatz</td></tr>
<tr><td>5.</td><td colspan="12">Wie viele Stunden arbeiten Sie in einer typischen Woche?</td></tr>
<tr><td></td><td colspan="12">Schieberegler: min: 5 Std; max: 50 Std; Stufen: 0,5 Std</td></tr>
<tr><td>6.</td><td colspan="12">Wer nutzt Ihren Arbeitsplatz (Schreibtisch, PC)?</td></tr>
<tr><td></td><td colspan="6">Nur Sie</td><td colspan="6">In Ihrer Abwesenheit auch Kolleg*innen</td></tr>
<tr><td>7.</td><td colspan="12">Wie viele Kolleg*innen nutzen gleichzeitig mit Ihnen Ihr Büro?</td></tr>
<tr><td></td><td colspan="3">Nur Sie</td><td colspan="3">Sie + 1 Kolleg*in</td><td colspan="3">Sie + 2 Kolleg*innen</td><td colspan="3">Sie + 3 oder mehr Kolleg*innen</td></tr>
<tr><td colspan="13">Sitzverhalten
Persönlich – Diese Fragen beziehen sich auf einen typischen Arbeitstag.</td></tr>
<tr><td>8.</td><td colspan="12">Wie viele Stunden arbeiten Sie an einem gewöhnlichen Arbeitstag?</td></tr>
<tr><td></td><td colspan="12">Schieberegler: min: 1 Std.; max: 10 Std; Stufen: 0,25 Std</td></tr>
</table>

9.	Wie viel Zeit verbringen Sie während Ihrer Arbeitszeit an einem gewöhnlichen Arbeitstag **im Sitzen**?					
	Schieberegler: min: 0 Std.; max: 10 Std; Stufen: 0,25 Std					
10.	Wie verteilt sich - **durchschnittlich** gesehen - diese Zeit im Sitzen **prozentual** auf folgende Tätigkeiten?<br>*Die Summe muss gleich 100 % oder 0 % sein!*					
	PC-Arbeit (auch Videokonferenzen)	*Schieberegler: min: 0 %; max: 100 %; Stufen: 5 %*				
	Telefonate	*Schieberegler: min: 0 %; max: 100 %; Stufen: 5 %*				
	Besprechung mit Kolleg*innen	*Schieberegler: min: 0 %; max: 100 %; Stufen: 5 %*				
	Lesen/Schreiben auf Papier	*Schieberegler: min: 0 %; max: 100 %; Stufen: 5 %*				
	Kurzzeitpausen	*Schieberegler: min: 0 %; max: 100 %; Stufen: 5 %*				
	Andere Tätigkeiten	*Schieberegler: min: 0 %; max: 100 %; Stufen: 5 %*				
11.	**Wie oft unterbrechen** Sie für gewöhnlich die sitzende Tätigkeit **pro Stunde**?<br>Jedes noch so kurze Aufstehen zählt bereits als Unterbrechung des Sitzens.					
	0x	1x	2x	3x	4x	5x oder mehr
12.	Seit der COVID-19-Pandemie und den damit einhergehenden Maßnahmen, unterbrechen Sie Ihre sitzenden Tätigkeiten pro Stunde…					
	Weniger häufig	Gleich häufig	Häufiger			
13.	Seit der COVID-19-Pandemie und den damit einhergehenden Maßnahmen, ist die Zeit, die Sie im Sitzen am Arbeitsplatz verbringen…					
	Weniger geworden	Gleich geblieben	Mehr geworden			
14.	**Wie oft wechseln** Sie für gewöhnlich **im Sitzen** Ihre Position **pro Stunde** (z.B. um das Gewicht zu verlagern, sich zu dehnen)?					
	0x	1x	2x	3x	4x	5x oder mehr
	**Stehverhalten**<br>Persönlich					
15.	Wie viel Zeit verbringen Sie während Ihrer Arbeitszeit an einem gewöhnlichen Arbeitstag **im Stehen**?					
	Schieberegler: min: 0 Std.; max: 10 Std; Stufen: 0,25 Std					
16.	Wie verteilt sich - **durchschnittlich** gesehen - diese Zeit im Stehen **prozentual** auf folgende Tätigkeiten?<br>*Die Summe muss gleich 100 % oder 0 % sein!*					
	PC-Arbeit (auch Videokonferenzen)	*Schieberegler: min: 0 %; max: 100 %; Stufen: 5 %*				
	Telefonate	*Schieberegler: min: 0 %; max: 100 %; Stufen: 5 %*				
	Besprechung mit Kolleg*innen	*Schieberegler: min: 0 %; max: 100 %; Stufen: 5 %*				
	Lesen/Schreiben auf Papier	*Schieberegler: min: 0 %; max: 100 %; Stufen: 5 %*				
	Kurzzeitpausen	*Schieberegler: min: 0 %; max: 100 %; Stufen: 5 %*				
	Andere Tätigkeiten	*Schieberegler: min: 0 %; max: 100 %; Stufen: 5 %*				

17.	Welche Angebote und Maßnahmen würden Sie sich im Arbeitsalltag wünschen, um Ihre Zeit im Sitzen zu reduzieren?<br>*Multiple Choice und Freitext möglich*
	Regelmäßige Erinnerungen um Aufzustehen durch eine App/SMS
	Regelmäßige Erinnerungen um Aufzustehen durch POP-UP/Mail am PC
	Verwendung eines Stehpultes
	Stehmeeting (bis 5 Teilnehmer, kurze Dauer, Raum mit Konferenztisch in Stehhöhe)
	Drucker, Abfalleimer etc. außerhalb der Reichweite im Sitzen
	Telefonaufkleber, der daran erinnert, Telefonate im Stehen zu führen
	Beratung oder Information über einfache Mittel zur Sitzunterbrechung
	Angeleitete Bewegungsvideos am Arbeitsplatz
	Keine Angebote erwünscht
	Ihre Ideen:

18.	Wie würde es Ihre Vorgesetzte oder Ihr Vorgesetzter vermutlich bewerten, wenn Sie an Ihrem Arbeitsplatz stünden?					
	Negativ	Eher negativ	Neutral	Eher positiv	Positiv	Weiß nicht

Sitzverhalten
Arbeitsweg

19.	Wie legen Sie Ihren Arbeitsweg üblicherweise **im Sommerhalbjahr** zurück?				
	PKW/Motorrad/Roller	Immer	Überwiegend	Selten	Nie
	Bus/Bahn	Immer	Überwiegend	Selten	Nie
	E-Bike	Immer	Überwiegend	Selten	Nie
	Fahrrad	Immer	Überwiegend	Selten	Nie
	Zu Fuß	Immer	Überwiegend	Selten	Nie

20.	Wie viel Zeit nimmt Ihr einfacher Arbeitsweg **im Sommerhalbjahr** in Anspruch?
	Schieberegler: min: 0 min.; max: 180 min; Stufen: 5 min
21.	Wie viel Zeit Ihres einfachen Arbeitswegs **im Sommerhalbjahr** verbringen Sie **im Sitzen; E-Bike und Fahrradzeiten ausgenommen**?
	Schieberegler: min: 0 min.; max: 180 min; Stufen: 5 min

22.	Legen Sie Ihren Arbeitsweg im Winterhalbjahr anders als im Sommerhalbjahr zurück?	
	Ja	Nein

23.	Wie legen Sie Ihren Arbeitsweg üblicherweise **im Winterhalbjahr** zurück?				
	PKW/Motorrad/Roller	Immer	Überwiegend	Selten	Nie
	Bus/Bahn	Immer	Überwiegend	Selten	Nie
	E-Bike	Immer	Überwiegend	Selten	Nie
	Fahrrad	Immer	Überwiegend	Selten	Nie
	Zu Fuß	Immer	Überwiegend	Selten	Nie

24.	Wie viel Zeit nimmt Ihr einfacher Arbeitsweg **im Winterhalbjahr** in Anspruch?
	Schieberegler: min: 0 min.; max: 180 min; Stufen: 5 min
25.	Wie viel Zeit Ihres einfachen Arbeitswegs **im Winterhalbjahr** verbringen Sie **im Sitzen; E-Bike und Fahrradzeiten ausgenommen**?
	Schieberegler: min: 0 min.; max: 180 min; Stufen: 5 min

Demographie

32.	Welchem Geschlecht fühlen Sie sich zugehörig?	
Weiblich	Männlich	Divers

33.	Wie alt sind Sie?								
≤ 20	21 - 25	26 - 30	31 - 35	36 - 40	41 - 45	46 - 50	51 - 55	56 - 60	> 60

34.	Was ist Ihr höchster Bildungsabschluss?
	Kein Abschluss
	Mittelschulabschluss (Hauptschulabschluss, Volksschulabschluss)
	Realschulabschluss (Mittlere Reife)
	Fachhochschulreife
	Allgemeine oder fachgebundene Hochschulreife/Abitur
	Berufsausbildung (z.B. Lehre, Berufsfachschule)
	Bachelor an einer (Fach-)Hochschule
	Fachhochschulabschluss (z.B. Diplom, Master)
	Universitätsabschluss (z.B. Master, Magister, Diplom, Staatsexamen)
	Promotion
	Anderer Abschluss

Die Beantwortung folgender Fragen ist **freiwillig**, wäre jedoch für die **wissenschaftliche Auswertung sehr hilfreich**.
Größe und Gewicht werden **nicht gespeichert**, sind jedoch für die Berechnung des **Body-Mass-Index (BMI)** erforderlich.

35.	Wie groß sind Sie?
	_ _ _ cm

36.	Wie viel wiegen Sie?
	_ _ _ kg

37.	Hatten Sie im letzten Jahr Beschwerden hinsichtlich körperlicher und psychischer Gesundheit?
Ja	Nein

38.	Welche Beschwerden hatten Sie hinsichtlich körperlicher und psychischer Gesundheit im letzten Jahr?

Onlinematerial 2 Flussdiagramm der Studienpopulation

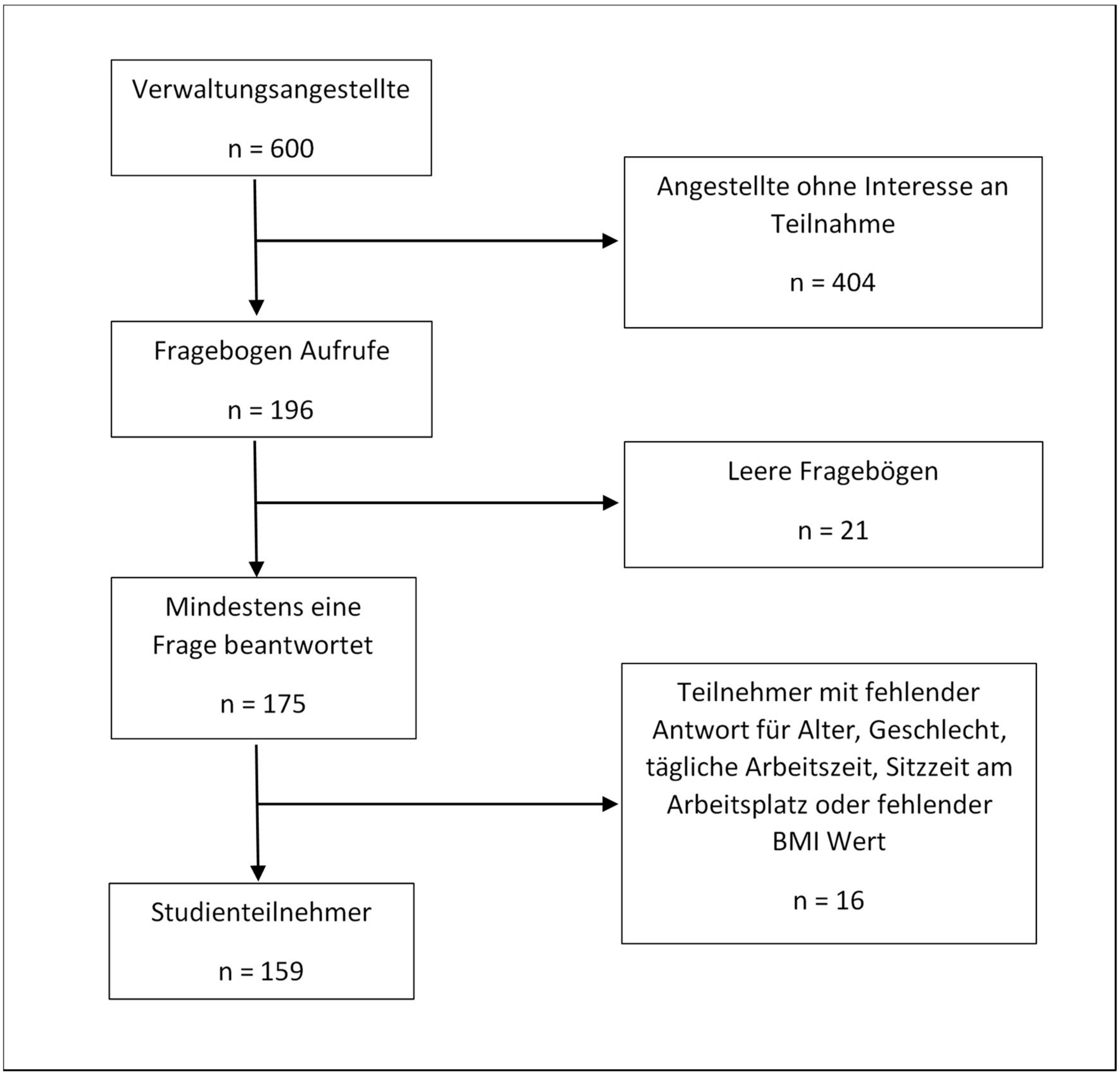

Onlinematerial 3 Vorhandenes Wissen der Teilnehmenden über die Auswirkungen von sedentärem Verhalten auf die Gesundheit stratifiziert nach Bildungsgruppe. n = 156

Bildungsgruppe	Muskel- und Skelettsystem	Körperliche Fitness	Übergewicht	Herz-Kreislauf-System	Typ 2 Diabetes	Krebs	Weiß nicht
Niedrig & Andere *(n = 35)*	91,4 % (32)	71,4 % (25)	77,1 % (27)	57,1 % (20)	22,9 % (8)	2,9 % (1)	2,9 % (1)
Mittel *(n = 48)*	98,0 % (47)	83,3 % (40)	72,9 % (35)	50,0 % (24)	20,8 % (10)	4,2 % (2)	0,0 % (0)
Hoch *(n = 73)*	100,0 % (73)	78,1 % (57)	78,1 % (57)	65,8 % (48)	26,0 % (19)	5,5 % (4)	0,0 % (0)

Onlinematerial 4 Gewünschte Interventionen der Teilnehmenden stratifiziert nach Bildungsgruppe. n = 156

	Bildungsgruppe		
	Niedrig & Andere (n=35)	Mittel (n=48)	Hoch (n=73)
Stehpult	54,3 % (19)	70,8 % (34)	68,5 % (50)
Stehmeeting	17,1 % (6)	39,6 % (19)	52,1 % (38)
Pop-up	20,0 % (7)	20,8 % (10)	32,9 % (24)
Bewegungsvideos	22,9 % (8)	10,4 % (5)	24,7 % (18)
Reichweite	25,7 % (9)	16,7 % (8)	19,2 % (14)
Beratung oder Information	28,6 % (10)	16,7 % (8)	16,4 % (12)
Sitz-Steh-Schreibtisch	17,1 % (6)	22,9 % (11)	13,7 % (10)
App	5,7 % (2)	8,3 % (4)	17,8 % (13)
Telefonaufkleber	0,0 % (0)	8,3 % (4)	5,5 % (4)
Betriebliche Angebote	8,6 % (3)	2,1 % (1)	1,4 % (1)
Angebote für Teams	0,0 % (0)	0,0 % (0)	4,1 % (3)
Andere	0,0 % (0)	0,0 % (0)	6,8 % (5)
Keine Angebote	8,6 % (3)	4,2 % (2)	5,5 % (4)

Onlinematerial 5 Gewünschte Interventionen der Teilnehmenden stratifiziert nach Altersgruppe. n = 159

	18 – 30 Jahre (n=38)	31-40 Jahre (n=39)	41 – 50 Jahre (n=31)	> 50 Jahre (n=51)
Stehpult	73,7 % (28)	79,5 % (31)	61,3 % (19)	54,9 % (28)
Stehmeeting	26,3 % (10)	43,6 % (17)	51,6 % (16)	41,2 % (21)
Pop-up	34,2 % (13)	30,8 % (12)	22,6 % (7)	21,6 % (11)
Bewegungsvideos	15,8 % (6)	28,2 % (11)	12,9 % (4)	21,6 % (11)
Reichweite	18,4 % (7)	17,9 % (7)	19,4 % (6)	23,5 % (12)
Beratung oder Information	15,8 % (6)	17,9 % (7)	29,0 % (9)	17,6 % (9)
Sitz-Steh-Schreibtisch	31,6 % (12)	23,1 % (9)	6,5 % (2)	7,8 % (4)
App	5,3 % (2)	7,7 % (3)	19,4 % (6)	17,6 % (9)
Telefonaufkleber	0,0 % (0)	2,6 % (1)	9,7 % (3)	7,8 % (4)
Betriebliche Angebote	2,6 % (1)	2,6 % (1)	3,2 % (1)	3,9 % (2)
Angebote für Teams	2,6 % (1)	2,6 % (1)	3,2 % (1)	0,0 % (0)
Andere	0,0 % (0)	0,0 % (0)	3,2 % (1)	7,8 % (4)
Keine Angebote	0,0 % (0)	0,0 % (0)	6,5 % (2)	13,7 % (7)
